# Wireless Systems and Networks in the IoT

**DOI:** 10.3390/s20082279

**Published:** 2020-04-17

**Authors:** Damianos Gavalas, Modestos Stavrakis, Periklis Chatzimisios, Zhichao Cao, Xiaolong Zheng

**Affiliations:** 1 Department of Product and Systems Design Engineering, University of the Aegean, 84100 Syros, Greece; modestos@aegean.gr; 2 Department of Science and Technology, International Hellenic University, 57001 Thessaloniki, Greece; pchatzimisios@ihu.gr; 3 School of Software, Tsinghua University, Beijing 100084, China; caozcthu@gmail.com; 4 School of Computer Science, Beijing University of Posts and Telecommunications, Beijing 100876, China; zhengxiaolong@bupt.edu.cn

**Keywords:** wireless systems, wireless networks, IoT, clock error compensation, channel prediction, RFID, backscatter communications, resource allocation, LoRaWAN, key generation

## Abstract

This Special Issue is focused on breakthrough developments in the field of Wireless Systems and Networks in the IoT. The selected contributions report current scientific progress in a wide range of topics covering clock error compensation in sensor networks, backscatter communication networks, Radio-Frequency Identification (RFID)-based inventory management, resource allocation in Long-Term Evolution (LTE)/LTE-A, (Long Range Wide-Area Network (LoRaWAN) modeling and key generation for the IoT.

## 1. Introduction

This Special Issue primarily solicits selected papers presented at the International Workshops collocated with the 2019 International Conference on Embedded Wireless Systems and Networks (EWSN 2019), held on 25 February 2019 in Beijing, China. EWSN is a highly selective annual forum for presenting research results in the field of networked embedded systems, broadly defined, including, for example, wireless sensor networks, Internet of Things, or cyber-physical systems. The workshops collocated with the EWSN 2019 have been the following:

- Workshop on Recent Advances in Wireless Coexistence for Heterogeneous IoT (CoWireless);
- 2nd International Workshop on Crowd Intelligence for Smart Cities: Technology and Applications (CISC 2019);
- 1st International Workshop on 6LoWPAN for the Internet of Things (6LoWPAN);
- Workshop on Security, Reliability, and Resilience in Wireless Sensor Networks (WSRRWSN);
- 1st Workshop on Low Power Wide Area Networks for Internet of Things (LPNET);
- 1st International Workshop on Distributed Fog Services Design (DFSD 2019).

The Special Issue also solicits extended versions of selected EWSN 2019 poster session papers as well as independent submissions. The theme of these contributions involved a broad range of topics pertaining to the area of wireless systems and networks in the IoT. The topics of interest included but have not been limited to:

- Architecture, design, implementation, and measurement of wireless coexistence for heterogeneous IoT systems;
- Cross-frequency communication in IoT;
- Cross-technology communication and interference;
- Big data analytics for IoT wireless networks and systems;
- 6LoWPAN for the Internet of Things;
- Security, reliability, and resilience in IoT communications;
- Low power wide area networks for Internet of Things, e.g., Long Range (LoRa), NarrowBand-Internet of Things (ΝΒ-ΙοΤ);
- Cloud computing and fog computing IoT wireless networks and systems.

## 2. Submissions, Review Process, Summary of Contributions

The special issue of the MDPI Sensors on ‘‘Wireless Systems and Networks in the IoT” has attracted numerous submissions. Following a rigorous review process, seven outstanding papers have been finally selected for inclusion in the special issue. Each paper received three reviews from independent experts. The accepted papers cover a wide spectrum of research topics in the broader area of the special issue.

The first paper presents a method of clock error compensation in sensor networks in order to provide a reference time for a wireless sensor network, which comprises the basis for real-time interaction, collaborative processing of sensor node information, and scheduling of network time [1]. The proposed method is based on a cyclic symmetry algorithm to compensate for the cumulative error of clock synchronization in the sensor network. The experimental results demonstrate that the compensation method significantly reduces the communication delay rate of the sensor network and improves its communication efficiency.

The second article proposes a channel prediction scheme for backscatter networks [2]. The prediction module predicts the channel quality at the next moment employing an algorithm based on the back-propagation neural network. Experimental and simulation results indicate the improved performance of the prediction mechanism in comparison to existing research with respect to switching channel time and overall throughput.

The third paper introduces a system for the efficient management and positioning of chemicals in chemical warehouses [3]. The proposed RF-Detector system combines robotics and Radio-Frequency Identification (RFID) technology to detect the remaining amount and localize chemicals in an autonomous fashion. The experimental results show that RF-Detector achieves high detection and positioning accuracy.

The fourth paper proposes a fast and reliable method for burst data transmission by fragmenting large data packets into blocks [4]. The paper also introduces a burst transmission mechanism to optimize the EPC Class-1 Generation-2 (EPC C1G2) communication procedure for burst transmission when there are critical and emergency data to be transmitted. Experimental results indicate that the proposed scheme significantly outperforms existing methods and that the goodput is close to the theoretically optimal value under different energy-harvesting and channel conditions.

The fifth paper introduces two uplink scheduling algorithms for Machine-to-Machine (M2M) devices over LTE [5]. Both algorithms take into consideration the channel quality when allocating resource blocks to devices, thus reducing the number of resource blocks necessary to send data, and consequently, reducing the energy consumed by devices. Simulation results show that the proposed schedulers outperform the round-robin scheduler in terms of energy efficiency and have better cell spectral efficiency.

The sixth paper introduces an accurate mathematical model of data transmission in LoRaWAN networks [6]. In contrast to previous work, the proposed model allows the estimation of important performance indices as the maximal affordable load, packet loss ratio, and delivery time distribution. The model can be used for performance evaluation, network planning, and selection of modulation and coding schemes.

The last paper proposes a signal strength exchange (SSE) system as an efficient key generation system and a synchronized quantization method as a part of the SSE system that synchronizes data blocks in the quantization phase [7]. The performance analysis at the IoT devices equipped with IEEE 802.11 radio demonstrates that the SSE system is more efficient in terms of computing time and communication overhead than existing systems.
